# Increasing the Difference in Decision Making for Oneself and for Others by Stimulating the Right Temporoparietal Junction

**DOI:** 10.3389/fpsyg.2019.00185

**Published:** 2019-02-06

**Authors:** Yinling Zhang, Siyang Chen, Xinmu Hu, Xiaoqin Mai

**Affiliations:** Department of Psychology, Renmin University of China, Beijing, China

**Keywords:** social neuroscience, decision making, self-other decision making, temporoparietal junction (TPJ), transcranial direct current stimulation (tDCS)

## Abstract

The right temporoparietal junction (rTPJ) has been thought to be associated with the difference in self-other decision making. In the present study, using noninvasive transcranial direct current stimulation (tDCS), we examined whether stimulating the rTPJ could modulate the self-other decision-making difference. We found that after receiving anodal stimulation of the rTPJ, participants were more likely to choose a high-value item for others than for themselves in the situations where the win probability of the high-value item was equal to or greater than that of a low-value item, indicating that elevating the cortical excitability of the rTPJ might increase the self-other decision-making difference in certain decision contexts. Our results suggest that decision making for others depends on neural activity in the rTPJ and regulation of the excitability of the rTPJ can influence the self-other decision-making difference.

## Introduction

In our social lives, we not only need to make decisions for ourselves but also often anticipate or make decisions for others. Indeed, decision making for others is the main content of social activities such as finance, medical treatment, consulting, and management, and acting for others in making risky decisions has gradually become an integral part of people’s social and economic lives. In recent years, the difference in self-other decision-making has attracted the attention of various researchers, and some research results and theories have emerged. However, few studies have directly explored the brain mechanisms of the difference in decision making between oneself and others.

While, some studies in the field of risky decision making have examined the self-other decision-making difference, the results are inconsistent. Hsee and Weber (1997) found that people estimated that others were more likely than themselves to choose uncertain risk options, regardless of whether the choice was between gains or between losses. Using a risk decision-making task with a gain frame, Stone et al. (2002) found that anticipated regret increased risk aversion, but they found no significant difference between decision making for oneself and decision making for friends. Fernandez-Duque and Wifall (2007) compared participates’ gambling behavior for themselves and advice giving to others regarding gambling, and found that participants showed more risk-taking tendencies in making decisions for themselves and relied less on objective probabilities when giving advice to others. Such inconsistencies arise because risk preference may be modulated by the gain/loss frame and risk probability (Liu et al., 2010, 2014; Duan et al., 2012). One study has found that people are less risk averse in a gain situation and less risk seeking in a loss situation when making decisions for a stranger than when making decisions for themselves (Zhang et al., 2017). These findings suggest that we cannot generally say whether people are more risk adverse or more risk seeking when making decisions for others than when making decisions for themselves. Rather, we must consider the contextual characteristics of decision tasks.

Researchers have also proposed some theoretical explanations for the inconsistency of risk preference in self-other decision making. For example, Loewenstein et al. (2001) propose the risk-as-feelings hypothesis that people’s response to risk anticipation is influenced by cognitive assessment and emotional response, with emotional response playing a decisive role in this process. The discrepancy between making decisions for oneself and predicting others’ decision making is due to the ”vividness” of others. The more vivid the other person is, the stronger the emotional response, which will affect the distinction in risk preference. Beisswanger et al. (2003) believe that compared with decision making for oneself, decision making on behalf of friends is associated with less anticipation of negative-emotion involvement and thus less risk aversion. Fernandez-Duque and Wifall (2007) found asymmetry in executor-observer risk decision making and proposed that when deciding for themselves, people rely more on empirical systems involving emotional and intuitive processing than on rational systems involving logical and analytical processing. This has been supported by some brain imaging studies, and the difference in decision making for oneself and that for others is reflected in the activation of the brain areas related to the emotional/empirical system and the cognitive/rational system. That is, decision making for oneself is more sensitive to rewards and perceived risks than decision making for others, as reflected in the greater involvement of the reward system and emotional-related brain areas, such as the ventral striatum, anterior cingulate gyrus, and amygdala (Albrecht et al., 2011; Jung et al., 2013). In contrast, decision making for others involves additional mentalizing and cognitive processing, as reflected in the activation of related brain areas, such as the temporoparietal junction (TPJ) and the medial prefrontal cortex (Janowski et al., 2013; Jung et al., 2013).

The right TPJ is a key brain area involved in processing different perspectives, reflecting and speculating mental states (Murray et al., 2012; Schurz et al., 2014, 2015; Krall et al., 2016; Mai et al., 2016). It is also an important part of other-processing networks and plays a role in promoting self-other distinction (Jardri et al., 2011; Venkatasubramanian et al., 2011; Murray et al., 2015; Steinbeis, 2016). Although relatively few studies have directly examined the brain mechanisms of the self-other decision-making difference, some studies have found that the TPJ plays an important role in this difference (Janowski et al., 2013; Jung et al., 2013; Ogawa et al., 2018). In an fMRI study, Jung et al. (2013) used a gambling task to directly compare the differences in brain activity when making risky decisions for oneself and for others. They found that different neural processes were involved in making risky decisions for oneself and for others; specifically, the reward system was more active in decision making for oneself, while the TPJ was more active in decision making for others. Ogawa et al. (2018) used a classical Theory-of-Mind task in an fMRI study and identified the rTPJ associated with cognitive perspective taking. They then examined whether activity in the identified rTPJ during the risky decision task (i.e., lottery-choice task) was modulated by the parameters of the behavioral-choice model, and found that rTPJ activity in the Other condition (i.e., decision making for an anonymous other) was modulated by the difference in expected value of the two lottery options, suggesting that individuals’ cognitive perspective taking operates in a more risk-neutral manner when making decision for others.

The transcranial direct current stimulation (tDCS) is a noninvasive brain stimulation technique, by which researchers can explore the casual relationship between neural activity in specific brain regions and cognitive function. It contains two electrodes, cathode and anode, which act on the cerebral cortex with weak current and regulate the activity of cerebral cortical nerve cells. Generally, the stimulation of anode electrode enhances the cortical excitability (i.e., anodal-excitation effect), while the cathode electrode causes an inhibition effect. The former is quite stable in cognitive studies (Jacobson et al., 2012). The tDCS has several advantages over other brain stimulation techniques. It is noninvasive, painless, safe, and easy to administer. The equipment is cheap and easily portable.

The purpose of the present study was to use tDCS to examine whether directly stimulating the rTPJ could modulate the self-other decision-making difference. In the present study, we used the gambling task adapted from the study of Jung et al. (2013). Considering that the decision context might affect the self-other decision-making difference, three decision situations were created in this task through two options in terms of value (high value and low value) and the probability of winning. The three decision situations are high-value option disadvantage, equal probability, and high-value option advantage. In the situation of high-value option disadvantage, choosing the high-value option is irrational and risky due to the high probability of loss; in the equal probability situation, choosing the high-value option is risky because of the high variability; and in the situation of high-value option advantage, choosing the high-value option is rational due to the high probability of winning. We hypothesized that elevating the cortical excitability of the rTPJ through anodal stimulation might increase the difference between decision making for oneself and that for others by inducing people to be more rational in making decisions for others; in contrast, inhibiting the excitability of the rTPJ through the cathodal stimulation was hypothesized have the opposite effect. Therefore, in the situations of high-value option disadvantage and equal probability, the anodal stimulation of the rTPJ would reduce the choice of the irrational or high-risk high-value option in decision making for others relative to decision making for oneself; in the situation of high-value option advantage, the anodal stimulation of the rTPJ would increase the choice of the rational high-value option in decision making for others.

## Materials and Methods

### Participants

Seventy-five adults (mean age 22.3 ± 1.7 years, 18 males) participated in this study as paid volunteers. They were randomly assigned to three groups of 25 participants each: the anodal, cathodal, or control sham. All participants were right-handed and had normal or corrected-to-normal vision. None of them reported a history of psychiatric or neurological disorders or a family history of epilepsy or a personal history of epilepsy. This study was carried out in accordance with the recommendations of the ethics committee of Department of Psychology at Renmin University of China. All participants gave written informed consent in accordance with the Declaration of Helsinki. The protocol was approved by the same ethics committee.

### Experimental Procedure

At the beginning of the experiment, each participant was informed to play the gambling game for him- or herself (self condition) and a stranger (other condition). They were given the phone numbers of 10 strangers and were asked to choose one of them. They would make decisions for the selected stranger in the following gambling game. In the condition of decision-making for oneself, each participant had 100 initial game points, and they were informed that the final game points were related to their own reward. In the condition of decision-making for others, the selected stranger also had 100 initial game points. Participants made decisions on behalf of the strangers and were told that the points they won for others would be converted into money and transferred to the stranger’s phone account, which means making decisions for others has nothing to do with the interests of the participants themselves.

**FIGURE 1:**
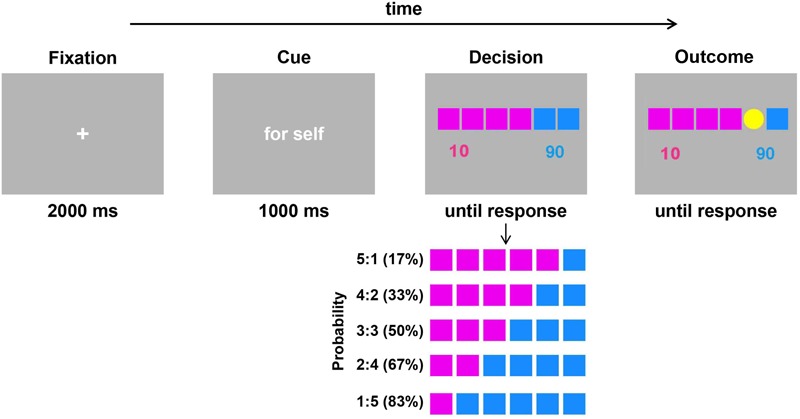
Schematic illustration of a single trial of the gambling task (adapted from Jung et al., 2013). Each trial began with a fixation cross, and a cue (“for self” or “for other”) then reminded participants whether the decision was for themselves or for a stranger. During the decision phase, there were five probability conditions for the two fixed value options. The percentage in parentheses represents the win probability of the blue square. Participants were asked to select one of the two colors (pink or blue) by pressing the “F” or “J” key. The outcome was presented after participants made responses.

The experimental task was adapted from the gambling task designed by Jung et al. (2013) to examine the distinction between decision making for oneself and decision making for others. As illustrated in Figure 1, each trial began with a fixation cross presented on the screen for 2000 ms. Then, a word “for self” or “for other” in Chinese appeared for 1000 ms, which cued the participant to make decisions for themselves or to make decisions for others. Afterward, six squares distinguished by pink and blue were presented, with the numbers 10 and 90 below the squares. The color of the squares represented the number of points (that is, value) that participants would win or lose: the value of the pink square was 10 points (low value), and the value of the blue square was 90 points (high value). The number of squares indicated the probability of winning or losing the corresponding number of points. There were five probability situations: 17, 33, 50, 67, and 83%. Each participant was asked to choose the pink or blue square by pressing the F or J key on the keyboard with their left or right index fingers. Pressing the F key represented selecting the pink square and pressing the J key represented selecting the blue square. After the participant responded, a coin represented by a yellow disk appeared randomly on any one of the six squares. If the coin appeared in the selected color square, the participant gained the corresponding value (add 10 or 90 points); if it did not appear in the selected color square, the participant lost the corresponding value (minus 10 points or 90 points). For example, when the participant chose the blue square and the coin appeared in one of the blue squares, he or she would get 90 game points. If it appeared in the pink square, he or she would lose 90 game points. The next trial began when the participant pressed the space key.

The formal experiment started after 4 trials of practice for each participant. There were 60 trials for the formal experiment, two decision roles (for themselves or others) and 5 high-value option probabilities (17, 33, 50, 67, and 83% chance of winning when choosing the blue square), resulting in 10 types of trials with 6 trials for each type. The order of the different types of trials was random. The stimuli were presented and behavioral data were recorded using E-Prime 2.0 software (PST, Inc., Pittsburgh, PA, United States).

### tDCS Protocol

The tDCS was delivered by a stimulator (DC-STIMULATOR MC, NeuroConn GmbH, Germany). The current stimulated the cerebral cortex through a pair of sponge coated electrodes (5 cm × 7 cm in size) soaked in saline. To stimulate the rTPJ, the anodal or cathodal electrode was placed between CP6 and C6 according to the international 10–20 EEG system and previous fMRI studies (Jurcak et al., 2007; Jung et al., 2013). This area covers the MNI coordinates [58, -66, 24] of the rTPJ reported in previous fMRI research (Jung et al., 2013). The reference electrode was placed on the left cheek. For the anodal and cathodal groups, a relatively weak direct current (1.5 mA) was continuously delivered for 20 min. For the reason of physical safety, current intensity is usually limited to 2 mA. If the duration is long enough, the excitability of cerebral cortex can reach more than 1 h after stimulation (Jacobson et al., 2012). In studies exploring the social cognitive function of the rTPJ, setting the current intensity at 1.5 mA is sufficient (Mai et al., 2016; Tang et al., 2018). For the sham group, although the electrode was placed over the rTPJ for 20 min, the current stimulation lasted only 15 s. The fade in and fade out time were both 15 s for each stimulation condition.

### Data Analysis

In this gambling task, the probability of the two options was variable, while the value attached to the option was fixed, and the difference multiplier of the value was greater than the maximum difference multiplier of the probability condition (i.e., the value of the blue/pink square was 90/10 greater than the 5/1). According to the equate-to-differentiate (ETD) strategy (Li, 2004; Li, 1994, Unpublished), individuals tend to focus on the high-value option for the invariant value dimension (i.e., the blue square of 90 points). Therefore, in the data analysis, we used the frequency of choosing the high-value option as the dependent variable.

In addition, the two value options were significantly different and fixed, and the win probability of each option varied between 17 and 83%. In previous research using this task, the risk level of the options was based only on the option values (Jung et al., 2013). However, the risk level of an option should take into account both the value and the probability. High-risk options thus generally refer to options with high value and a low probability of winning (Krain et al., 2006; Smith et al., 2009). According to the expected utility theory (von Neumann and Morgenstern, 1947), the rational choice in decision making is to choose the option with greatest expected utility. When the probability changes, the expected utility of the option also changes. The high-value option thus does not always represent the irrational risk-taking alternative to the rational choice. Therefore, in this study, we considered the size of value, the probability of winning, and the expected utility. According to the probability of winning of the high-value option, we divided them into three types of probability situations: high-value option with low probability/disadvantage situation (17% and 33%), equal probability situation (50%), and high-value option with high probability/advantage situation (67 and 83%). When the probability of wining the high-value option is lower (17 and 33%), the high-value option is the irrational, high-risk choice with a high probability of loss; when the probability of winning the high-value option is 50%, the high-value option is the high-risk choice; and when the probability of winning high-value option is greater (67% and 83%), the high-value option is the rational choice with a high probability of winning (see Table 1).

**Table 1:** Meaning of the options under the different probability situations.

Option	High-value option disadvantage (17% and 33%)	Equal probability (50%)	High-value option advantage (67% and 83%)
High value (90 points)	Irrational, high risk	High risk	Rational
Low value (10 points)	Rational, low risk	Low risk	Irrational

The frequency of choosing the high-value option was subjected to a two-way repeated-measures analysis of variance (ANOVA) with one between-subjects factor (tDCS groups: cathodal, sham, and anodal) and one within-subject factor (decision-maker role: for self and for other) evaluated for the three probability situations: high-value option disadvantage, equal probability, and high-value option advantage. The data were statistically analyzed using SPSS software (version 21.0, IBM Corp., Armonk, NY, United States).

## Results

**FIGURE 2:**
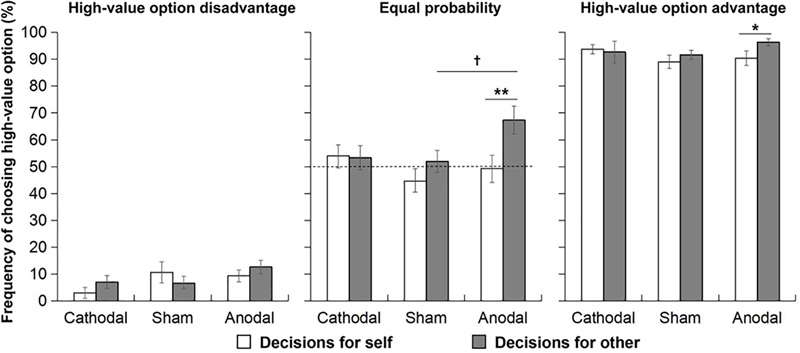
In three probability situations, the frequency of choosing the high-value option for oneself and others in the three tDCS groups. The dashed line represents risk-neutral behavior (choosing the high-value option in 50% of trials). Error bars indicate SEM. †*P* < 0.1; ^∗^*P* < 0.05; and ^∗∗^*P* < 0.01.

In the situation of high-value option disadvantage, the 3 (tDCS group: cathodal, sham, and anodal) × 2 (decision maker role: for self and for other) repeated-measures ANOVA did not show any significant main effect or interaction effect on the frequency of choosing the high-value option, indicating that there was no self-other difference in decision making in each tDCS group in the situation of high-value option disadvantage, i.e., when choosing the high-value option was irrational and risky (Figure 2A).

In the situation of equal probability, the main effect of decision-maker role was significant, *F*(1, 72) = 5.74, *p* = 0.019, $\eta_{p}^{2}$ = 0.07, indicating that the frequency of choosing the high-value option for others was higher than that for oneself. The interaction effect between decision-maker role and tDCS group was marginally significant (*F*(2, 72) = 2.48, *p* = 0.091, $\eta_{p}^{2}$ = 0.07). Pairwise comparisons were conducted to further examine the self-other difference in decision making for each tDCS group. The results showed that in the anodal group, the frequency of choosing the high-value option for others (*M* = 0.67, *SD* = 0.26) was significantly higher than that for oneself (*M* = 0.49, *SD* = 0.25, *p* = 0.003), but this self-other difference was not found for the cathodal and sham groups. In addition, to examine the difference in decision making among the three tDCS groups for each decision-maker role, a one-way ANOVA was conducted separately between decision making for oneself and decision making for others. The results showed that the frequency of choosing the high-value option was different among the three tDCS groups only for decision making for others (*F*(2, 72) = 3.35, *p* = 0.041, $\eta_{p}^{2}$ = 0.09). *Post-hoc* comparisons found that the frequency of choosing the high-value option for others was higher in the anodal group (*M* = 0.67, *SD* = 0.26) than in the sham group (*M* = 0.52, *SD* = 0.21), marginally significant, *p* = 0.067. Moreover, in the anodal group, the frequency of choosing the high-value option for others was more than 50% (*t* (24) = 3.32, *p* = 0.003, *d* = 0.92), see Figure 2B.

In the situation of high-value option advantage, only the main effect of decision-maker role was marginally significant (*p* = 0.093). Based on our hypothesis, we focused on the comparison between the “self” and “other” condition in each stimulation group. In order to avoiding missing some meaningful information due to just relying on omnibus *F*-test, we conducted pairwise comparisons to further examine the self-other difference in decision making for each tDCS group. The results showed that in the anodal group, the frequency of choosing the high-value option for others (*M* = 0.96, *SD* = 0.06) was significantly higher than that for oneself (*M* = 0.90, *SD* = 0.14, *p* = 0.024), but this self-other difference was not found in the cathodal and sham groups (see Figure 2C). In addition, one-way ANOVA was conducted to examine the difference in decision making among three tDCS groups for each decision-maker role, but the frequency of choosing the high-value option was not different among the three tDCS groups for both decision-maker roles.

## Discussion

The present study examined the effects of regulating the excitability in the rTPJ by tDCS on self-other differences in risky decision making. We found that in different situations of win probability, the participants behaved differently when we elevated the excitability of their rTPJ. Specifically, in the situation of high-value option disadvantage, in which choosing the high-value option was irrational and risky, exciting or inhibiting rTPJ activation did not influence decision making for oneself and others. Further, in the situation of equal probability, in which the high-value option had high risk, increasing the rTPJ excitability made participants more likely to choose the high-risk item for others (exceeding risk-neutral value 50%) than for themselves. This finding indicates that individuals are more adventurous when making decisions for others, which is contrary to our hypothesis. Finally, in the situation of high-value option advantage, in which the high-value item was the rational option, exciting the rTPJ made participants choose more high-value items for others than for themselves. It suggests that individuals are more rational when making decisions for others, which is consistent with the hypothesis. Overall, these results suggest that the rTPJ plays an important role in decision making for others and that regulation of the excitability of the rTPJ can influence the self-other decision-making difference.

When elevating the cortical excitability of participants’ rTPJ, the frequency of choosing the high-value option for others was significantly higher than that for oneself in the situations of high-value option advantage and equal probability, suggesting that the rTPJ is involved in self-other differences in decision making. When making decisions for others, individuals as decision-makers need to distinguish themselves from others, realize that they are making choice on behalf of others (i.e., change perspectives), and even consider the impact of potential outcomes on others (i.e., theory of mind). Brain imaging studies has also found that the TPJ is related to perspective taking (Santiesteban et al., 2012; Schurz et al., 2015) and theory of mind (Schurz et al., 2014; Krall et al., 2016; Mai et al., 2016). It is more active in making decisions for others (Jung et al., 2013).

However, the high-value potion is the rational choice in the high-value option advantage situation, but the high-risk choice in the equal probability situation. It indicated that participants made more rational decisions for others than for themselves in the high-value option advantage situation, while they made more risky decisions for others than for themselves in the equal probability situation. These results are contradictory with each other and partly contrary to our hypothesis. Nevertheless, if we ignore the context-dependent meaning of the option and focus on the option itself, we can find that the frequency of choosing high-value option with high potential gain and high potential loss values was in fact greater for others than for oneself in both situations. It seems to be caused by the weakening of the impact of the high potential loss (may be the aversion to potential loss) in the “other” condition. Jung et al. (2013) reported that right amygdala activation was positively correlated with individuals’ probability of choosing high-value items for themselves, while the activity of the left dorsomedial prefrontal cortex (DMPFC) was positively correlated with the probability of choosing high-value items for strangers. In addition, further analysis found a stronger functional connectivity between the rTPJ and the left DMPFC in decision making for others than that for oneself (Jung et al., 2013), while amygdala activation could be regulated by the DMPFC through the functional connectivity between these two brain areas (Banks et al., 2007; Phillips et al., 2008; Leiberg et al., 2011; Potvin et al., 2017). Brain imaging studies of risky decision making have found that the amygdala is not only sensitive to potential loss in decision making but also closely related to loss aversion (Bechara et al., 1999; De Martino et al., 2010; Sokolhessner et al., 2013; Phelps et al., 2014). Therefore, we believe that the TPJ may not only play a role in perspective taking and interpreting other’s mental state, but also may indirectly regulate some cognitive processes through its connection with the DMPFC. Moreover, the activation of the left DMPFC can negatively regulate amygdala activation, which inhibits the avoiding response to potential high losses of the high-value option and makes individuals more likely to choose it. Hence, we can explain the seemingly contradictory results in this way. In situations in which the win probability of the high-value item is equal to or greater than that of the low-value item, the anodal stimulation of the rTPJ may reduce the emotional response to the potential loss of the high-value option by increasing the inhibition of the DMPFC on the amygdala in decision making for others, and thus increase the frequency of choosing high-value items for others rather than for oneself.

In the high-value option disadvantage situation, anodal stimulation of the rTPJ did not increase the self-other decision-making difference. One explanation is that because the high-value item is the irrational and high-risk choice in this situation, a floor effect may arise owing to the low frequency of choosing the high-value item. Another possible explanation is that amygdala activity is strong when the win probability of the high-value item is low and the loss probability is high (i.e., the disadvantage situation for the high-value item), and although exogenous enhancement of rTPJ activity can promote the regulation of the amygdala by the DMPFC, the amygdala activity remains strong enough to induce the individuals to avoid options with a high probability of a large loss. Therefore, the role of anodic stimulation in the situation of high-value option disadvantage is not obvious.

We did not observe any cathodal effect on the participants’ decision making, which was inconsistent with our assumptions that cathodal stimulation of the rTPJ could reduce the difference in self-other decision making. This may be because there was no self-other decision-making difference in the sham group. Therefore, although inhibiting the rTPJ activation can reduce the difference in self-other decision making, it is impossible to show such a reduction in this study due to the floor effect. In addition, the inhibition effect of negative stimulation may usually be influenced by the initial activation state of neurons and the functional compensation of related brain regions in cognitive studies (Jacobson et al., 2012). The left TPJ (LTPJ) is also involved in theory of mind (Gallagher et al., 2000; Samson et al., 2004; Ye et al., 2015), and it is functionally connected with the prefrontal lobe in decision making for others (Janowski et al., 2013). Thus, the LTPJ might play a compensatory role in inhibiting the rTPJ, resulting in a lack of behavioral changes when the cortical excitability of the rTJP is inhibited.

The self-other difference of decisions was not shown in the sham group for each of the decision situations. Previous studies have reported inconsistent results regarding risk preference in self-other decision making, which might be caused by the different decision contexts (Hsee and Weber, 1997; Stone et al., 2002; Fernandez-Duque and Wifall, 2007). Some researchers have found that the self-other difference in risk decision-making can be affected by the gain/loss framework, the decision makers’ self-esteem level, and the mental distance between decision makers and others (Liu et al., 2010; Duan et al., 2012; Zhang et al., 2017, 2018). Thus, the gambling task we applied in the present study may not be sensitive enough to examine differences in decision-making between for oneself and for others when no stimulation is applied to the rTPJ. The lack of a self-other decision-making difference in the sham group may restrict our investigation of the role of cathodal stimulation. Therefore, to better understand the function of the rTPJ, in future research, it is necessary to consider the possible factors that influence the self-other decision-making difference and design a task by which we can observe the difference in decision making between for oneself and for others when no stimulation is applied to the rTPJ.

There are some limitations to our study. First, we designed the sham stimulation group as a control group in which participants did not receive effective stimulation. But the general effect of the anodal stimulation on cerebral cortex cannot be ruled out. To help us better understand the specific function of the rTPJ, in the future studies we can stimulate another brain region to serve as a control region, such as the occipital cortex which does not associate directly with decision making or some other regions which are responsible for decision making in general but not specifically for self-other differences in decisions. In addition, the manipulation of “others” is relatively rough compared to real life. Prior studies have found that similarities, relationships, and preferences for others relate to different neural systems (Guroglu et al., 2008; Krienen et al., 2010; Wang et al., 2012; Braams et al., 2014; Zhao et al., 2016). Some researchers believe that the fundamental difference between oneself and others is mental distance (Liu et al., 2014). Braams et al. (2014) compared neural activity when participants engaged in gambling tasks for themselves, good friends, and disliked others and found that brain areas associated with social information processing, such as the DMPFC and TPJ, showed greater activation with respect to the win or loss outcome of gambling for others than for themselves and friends. Therefore, to comprehensively examine the neural mechanisms of the self-other decision-making difference, further studies involving different mental distance between oneself and others are needed.

The present study validates the crucial role of the rTPJ in the self-other decision-making difference through elevating the cortical excitability suggesting that decision making for others depends on the neural activity in the rTPJ, which has important implications for us to understanding the function of the TPJ in self-other decision making. The TPJ may not only associate with perspective taking and theory of mind, but also indirectly regulate emotion responses through its functional connection with the medial prefrontal lobe. Combining the tDCS with other techniques that can record neural and peripheral physiological activity in future studies would help us further reveal the neural mechanism of decision making for others and understand specific economic phenomena (e.g., loss aversion) from the perspective of decision making for oneself and others.

## Author Contributions

XM and SC designed the study. SC, YZ, and XH collected and analyzed the data. YZ wrote the manuscript. XM and XH edited the manuscript. All authors approved the final version of the paper for submission.

## Conflict of Interest Statement

The authors declare that the research was conducted in the absence of any commercial or financial relationships that could be construed as a potential conflict of interest.
